# Thymoma calcification: Is it clinically meaningful?

**DOI:** 10.1186/1477-7819-9-95

**Published:** 2011-08-23

**Authors:** Kassem Harris, Dany Elsayegh, Basem Azab, Homam Alkaied, Michel Chalhoub

**Affiliations:** 1 Staten Island University Hospital, Department of medicine, 475 Seaview ave, Staten Island, NY 10305, USA

**Keywords:** thymoma, calcification, diagnosis, imaging, mediastinal

## Abstract

Among anterior mediastinal lesions, thymoma is the most common. Thymomas are tumors of thymic epithelial cell origin that are distinguished by inconsistent histological and biologic behavior. Chest imaging studies typically show a round or lobulated tumor in the anterior mediastinum. Calcifications in thymomas are classically punctuate or amorphous, positioned within the lesion. Chest computed tomography (CT) features suggesting higher risk thymoma consist of tumor heterogeneity, vascular involvement, lobulation, pulmonary nodules, lymphadenopathy, and pleural manifestations. Imaging findings have an imperfect ability to predict stage and prognosis for thymoma patients. Our objective is to highlight the clinical implications of thymoma calcifications on the diagnosis, clinical manifestation and prognosis. A pubmed and google search was performed using the following words: thymoma calcification, calcified thymus, mediastinal calcification, anterior mediastinal calcification, and calcified thymoma. After reviewing 370 articles, 32 eligible articles describing thymoma calcifications were found and included in this review. Although the presence of thymus calcifications was more common in patients with invasive thymomas, they were present in significant portion of non-invasive thymomas. The presence of calcifications was not a significant factor in differentiating between benign and malignant thymoma. As a result, the type, location, size or other characteristics of thymus gland calcifications were not relevant features in clinical and radiologic diagnosis of thymoma. The histopathological diagnosis is still the only possible way to confirm the neoplastic nature of thymoma. All types of thymomas should be evaluated and managed independently of the presence of calcifications.

## Introduction

The thymus gland is located in the anterior mediastinal compartment of the chest. It originates from the third and fourth branchial pouches and is composed from all three germinal layers. The thymus gland is mostly active in the pre-adolescent period and it the largest in size. It starts to coalesce and becomes completely atrophic with remnant adipose tissue by the late teens. Nonetheless, lymphopoiesis of the T cells continues during adult life. Tumors of the thymus gland are the most commonly encountered abnormal tissue growth of the anterior mediastinal compartment. Lymphatic and germinal tumors are the second most frequent. Thymic tumors are usually epithelial in origin, with low-grade neoplasm and slow growth rate. The propensity of a thymoma to be malignant is determined by the invasiveness of the thymoma. Thus, thymomas without local invasion are considered benign tumors. Other tumors include parathyroid, thyroid tissue, vascular, and mesenchymal tissue masses. Occasionally, thymic tumors can be found in the posterior mediastinum or other locations like lower neck. Calcification can occur in benign and malignant thymomas and up to 40 percent of thymomas present with some type of calcifications [1]. In this review, we will go over types of thymoma calcifications and their clinical significance.

### Epidemiology

Thymoma is a rare neoplasm that account for approximately 0.2-1.5% of all malignant neoplasms. Among the anterior mediastinal tumors, thymoma is the most frequent. It represents 20% of all mediastinal neoplasm in adults [2,3]. Thymoma usually affects patients in the middle age with no sex predominance. About 35 percent of thymic tumors are malignant with the exception of patients between the ages of 20 to 40 where cancers account for about half of thymic tumors. Thymoma occurs with increasing frequency as age increases. It goes from 3% at age of 20 to about 12% at ages 21 to 45 years. About 35 percent of thymic tumors occur after 46 years of age [4]. Men and women are equally affected [5]. Thymoma is usually discovered incidentally on chest imaging studies performed for other reasons. Approximately 50% of thymomas are asymptomatic at the time of diagnosis. Depending on study groups, the frequency of thymomas with calcification ranges from 10% to 41% [1,6] and in cases of invasive thymomas, it can go up to 54% [1]. Among other forms of calcifications found in thymoma, small foci of calcification are the most common. Thymoma with massive calcification is uncommon and when present, it is usually called dystrophic calcification.

### Clinical Presentation

Approximately half of patients are asymptomatic at the time of thymoma diagnosis and are incidental findings [7]. In general, an approximate of 55% of patients with benign mediastinal tumors is asymptomatic. Contrary, only about 15% of those in whom tumors are found to be malignant are asymptomatic. When symptomatic, systemic symptoms related to tumor-related syndromes like myasthenia gravis are predominant. Symptomatic thymomas are usually invasive with local compression. In other cases, dyspnea, cough and chest pain can be the presenting symptoms [8]. In the presence of an anterior mediastinal lesion, the diagnosis of thymoma is usually clinically suspected based on the presence of myasthenia gravis or other associated syndromes such as autoimmune or immune diseases, endocrine abnormalities and syndrome of inappropriate anti-diuretic hormone (SIADH) [9]. There were no previous reports correlating thymoma calcifications to any specific symptoms.

### Imaging Manifestations

As the configuration of the thymus gland changes with age, imaging is expected to change as well. In the pre-puberty period, the thymus attenuation on CT scan is proportionate to that of the adjacent musculature. The thymus lobes are usually separated with slight rotation to the left from the midline. As the thymus becomes infiltrated with fatty tissues, the attenuation starts to recede compared to surrounding musculature. After the age of 30, the thymus gland remnants appear as insular areas of low attenuation soft tissue. They are usually surrounded by higher attenuation fatty tissue. As we get older, the thymus gland is seen as a thin fibrous tissue with total deposition of fat. As the configuration of the thymus gland may change from person to person depending on various factors, the most dependable and concise radiologic way to evaluate the thymus is by its thickness.

Computed tomography (CT) scanning is the most admired test for the diagnosis of thymoma and other anterior mediastinal lesions. On computed tomography (CT) and magnetic resonance imaging (MRI), most thymomas appear as spherical or ovoid lesions [10]. They are usually well defined, lobulated, heterogeneous, and located in the anterior-superior mediastinum. Although most thymomas are homogenous attenuation lesions, they may appear as soft-tissue masses that contain regions of low attenuation, which could be secondary to necrosis. On CT imaging, the areas of calcification and hemorrhage are characterized by high attenuation densities [3,10]. Irregularities are usually the signs of local invasion and aggressive carcinomas [3,11]. The most reliable and meaningful measurements of the thymus are related to its thickness. Although the normal thickness before the age of 20 can be up to 1.8 cm, normal thymus thickness in adults should not exceed 1 cm. Larger size, irregular contours, necrotic or cystic areas, and foci of calcification, in addition to heterogeneous density on CT scan, are likely signs of invasive thymoma [12]. Calcification was found more notably in invasive thymoma [1,13].

In small series, all patients with thymoma and calcifications were malignant [13,14]. However, calcification per se cannot differentiate between benign and malignant thymomas [15]. In a study for CT scan and pathology correlation, Jung et al. identified thymoma calcifications in one third of patients with high risk thymoma and in 61% of patients with thymic carcinoma [16]. Thymoma may present as egg shell in the chest [17] and in some cases, it can be hard to differentiate from hemangioma [18]. At times, thymic cysts may present as ring-like calcified lesions. Rarely, linear calcifications in the cyst wall are found. The typical water density on the CT scan differentiates thymic cysts from thymoma. Other mediastinal pathologies may display calcifications. These lesions can have focal areas of calcifications upon diagnosis, like thymic carcinoma, mediastinal seminoma, thymic carcinoid tumors, and hemangioma [3,19]. Tumors of the sternum can present a challenge when considering the diagnosis of thymoma. Radiologically, vascular lesions and other bone tumors arising from the sternum may be comparable to thymoma, which make it difficult to differentiate between these tumors. Scattered calcifications have been reported in thymic amyloid tumors as well [20]. Moreover, mediastinal calcifications have been associated with other lesions like germ cell tumors, including mature teratoma, thyroid tumors, mesothelioma, lymphoma, and other anterior rare mediastinal lesions such as mesenchymal tumors [4]. In cases of lymphoma, calcifications are sporadic and they often occur at about 7-8 months after the onset of treatment [21]. The combinations of fluid, soft and fatty components, which may also include osseous or dental elements, support the diagnosis of mature teratoma [3]. Occasionally, mediastinal goiter can present with coarse, punctuate or ring like-calcifications.

Positron emission tomography (PET) scan can be helpful in the evaluation of tumor extension [22]. Thymic carcinoma has higher uptake than other better differentiated thymic epithelial tumors, as well as normal or hyperplastic thymus [23]. Using an SUV cut-off point of 5.0 high sensitivity (84.6%) and specificity (92.3%) can be achieved when trying to distinguish between thymic carcinoma and thymoma [24]. Finally, Thymomas uncommonly show calcifications at chest radiography, and when present, the patterns typically seen include curvilinear or punctuate calcifications [3,4,19]. Plain film can demonstrate amorphous, flocculent calcification. In some cases, rim calcified thymomas can be clearly identified on plain film. Thymoma with rim calcification is rare and this type of calcification has been described in association with benign cystic thymoma [4]. Table 1 summarizes the studies and reported cases describing the percentage of thymoma calcifications. The revised World Health Organization (WHO) in 2004 classified thymomas based on morphology and ratio of lymphocytes to epithelial cells. There were 6 categories, A, AB, and B1 are the low risk thymoma, and B2 and B3 are the high risk thymoma. The sixth category is the thymic carcinoma. Thymus calcification was more frequently seen in type B1, B2 and B3 thymomas. It was less frequent in type AB and in thymic carcinoma (previously classified as type C) [25,26]. A high degree of homogeneous enhancement has the propensity to indicate low risk thymoma (type A or AB).

**Table 1:** Summary of studies and reported cases describing the percentage of calcifications in patients with thymoma and thymic carcinoma

Reference	Noninvasive thymoma	Invasive thymoma	Thymic carcinoma
**Tomiyama et al**.	26	54	-
**Sone et al**.	0	40	-
**Doe et al**.	-	17	10
**Jung et al**.	-	33	61
**Priola et al**.	9	43	-
**Jeong et al**.	10	31	20

### Histopathology

Pathological diagnosis can be very difficult as there is no clear histological distinction between benign and malignant thymomas. Therefore, definite diagnosis requires surgical excision of the thymoma. Signs of malignancy are local invasion through the capsule and to the surrounding tissues. Malignant thymomas can invade the adjacent structures including vasculature and lymphatics. The Masaoka staging system of thymomas is the most commonly accepted system, with stage I when the tumor is encapsulated, and stage IV with evidence of metastatic spread. In general, a thymoma is deemed malignant when gross or microscopic tumor invasion, recurrence, or distant metastases are present [3]. Macroscopically, invasive thymomas may resemble to have an intact capsule, and histologic examination is required to confirm the diagnosis. On occasions, prominent macroscopic invasion may be apparent [27]. Larger size thymomas are more likely to exhibit calcifications, hemorrhage and cystic changes [27,28]. Oftentimes, Thymomas is diagnosed in association with other conditions, of which myasthenia gravis is the most common [3]. Calcification in thymomas in association with myasthenia gravis has been stated in 7-25% of patients [3,4,19]. However, diagnosis from only imaging findings is of limited value in differentiating the various histologic subtypes of thymomas [29].

### Management

Surgery is the mainstay of therapy, and all thymomas, except completely encapsulated stage I tumors, benefit from adjuvant radiation therapy [22]. Recurrence rates after surgical excision varies between 8 - 29% depending on the series and type of tumour [30]. Local invasion and distant tumor spread have been found to be the most significant factor for survival [31]. In cases of invasive thymoma, the leading causes of death are cardiorespiratory complications including cardiac tamponade.

**Figure 1:**
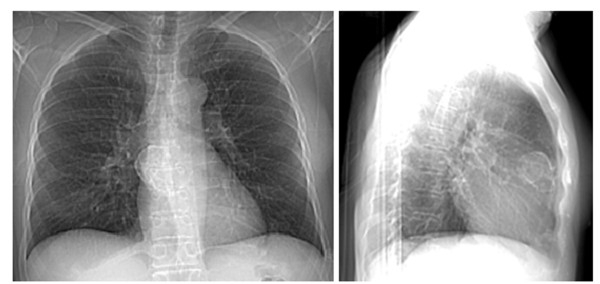
**Frontal chest radiograph shows a rim calcified lesion (left panel)**. Lateral chest radiography confirms that the rim calcified mass resides within the anterior mediastinum (right panel).

**Figure 2:**
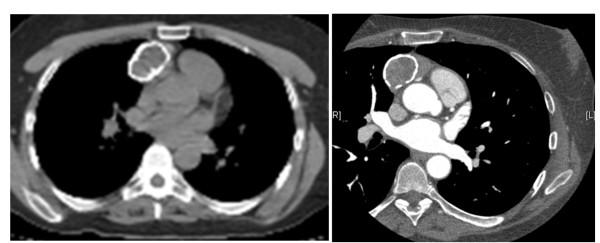
**Computed tomography (CT) scan showing a 3.9 cm rim calcified right anterior mediastinal mass**. CT with intravenous contrast (right panel) shows the mass in relation to mediastinal vasculature.

**Figure 3:**
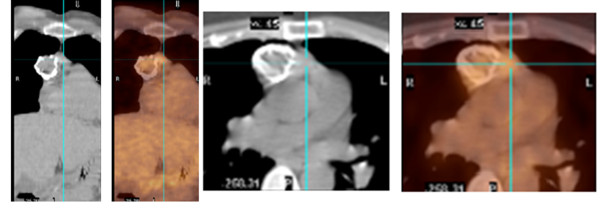
**PET/CT scan: 2 views showing the 3.9 cm rim calcified right anterior mediastinal mass which is FDG avid (SUV 6)**. The adjacent midline anterior mediastinal density is FDG avid as well (SUV 3.9).

**Figure 4:**
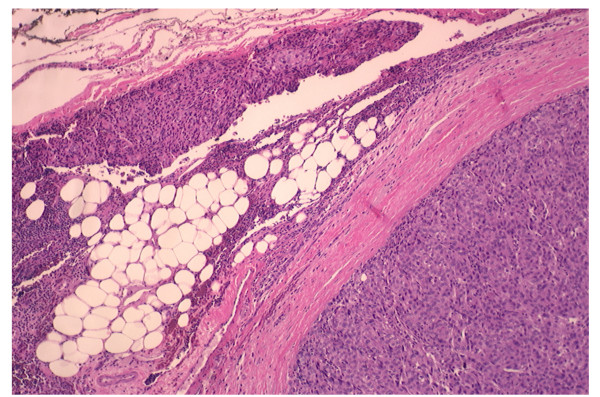
**Thymoma with lobular growth pattern and invasion into mediastinal fat (hematoxylin-eosin, original magnification X40)**.

The prognosis of patients with a thymoma is based on the tumor's gross characteristics at operation, not the histological appearance. In case of invasive carcinoma, the 15-year survival rate is 12.5 percent, and 5 years survival is about 35 percent. It is 47 percent for patients with a noninvasive thymoma [22]. Medullary and lymphocyte thymoma have the best survival, which is about 85 percent at 5 years [32]. Recently, we had a patient who presented with a plain chest film showing 5 × 2.5 cm anterior mediastinal mass which exhibits rim-like calcifications of the mass (Figure 1). Computed tomography (CT) of the chest showed 4 × 3 cm rim calcified minimally lobulated anterior mediastinal mass with predominantly solid internal contents with adjacent soft tissue lesion in the anterior mediastinum (Figure 2). The chest CT was followed by Positron emission tomography (PET) scan which showed a 3.9 cm rim calcified right anterior mediastinal mass. The mass was 18F-fluoro-2- deoxyglucose (FDG) avid with standard uptake value (SUV) of 6. The adjacent midline anterior mediastinal lesion was FDG avid with SUV of 3.9 as well, suspicious for biologic tumor activity (Figures 3). Robotic assisted surgery was performed with excision of the entire calcified lesion. The excised tissue was 10.4 × 5.6 × 2.8 cm calcified, lobulated tissue weighing 40 grams. Pathological diagnosis was consistent with thymoma, type B2, with microscopic transcapsular invasion (Figure 4). The surgical margin was negative and the thymoma was classified as modified Masaoka stage IIa.

## Conclusion

When anterior mediastinal lesion is suspected or diagnosed as thymoma, calcification doesn't seem to have any significantly meaningful diagnostic or prognostic value. All study groups were retrospective with small samples. Although the presence of thymus calcifications was more common in invasive thymomas, they were present in significant portion of non-invasive thymomas. The presence of calcifications was not useful in differentiating between benign and malignant thymoma. Even in cases that present as rim-calcified tumors, invasive thymomas are still possible and one should proceed with more sophisticated workup such as PET scan and subsequently, pathological diagnosis. Therefore, type, location, size or other characteristics of thymus gland calcifications were not important factors in clinical and radiologic diagnosis of thymoma. The histopathological diagnosis is still the gold standard to confirm the neoplastic nature of thymoma. All types of thymomas should be evaluated and managed independently of the presence of calcifications. Thymoma is a rare entity which precludes the initiation of large prospective trial.

## Competing interests

The authors declare that they have no competing interests.

## Authors' contributions

KH wrote the manuscript and carried out the research. DE, BA, HA, and MC reviewed the manuscript. All authors read and approved the final manuscript.
